# Atraumatic splenic rupture in young adult following cocaine use

**DOI:** 10.1016/j.ijscr.2019.10.081

**Published:** 2019-11-03

**Authors:** Joshua Lee Ramos, Michael Farr, Seung Hoon Shin, Nasim Ahmed

**Affiliations:** a Division of Trauma & Surgical Critical Care, Jersey Shore University Medical Center, Neptune, NJ, USA; b Department of Surgery, Monmouth Medical Center, Long Branch, NJ, USA

**Keywords:** Atraumatic splenic rupture, Splenomegaly, Cocaine

## Abstract

**Introduction:**

Splenic rupture most commonly results from a traumatic mechanism. Spontaneous splenic ruptures are a rare occurrence. This is an interesting and rare case of atraumatic splenic rupture (ASR) presented after sniffing cocaine.

**Presentation of case:**

The patient presented with abdominal pain and tenderness with low hemoglobin on admission. The computed tomography (CT) scan of the abdomen showed possible spleen rupture with hemoperitoneum. An emergent laparotomy and total splenectomy was performed. The pathology confirmed splenomegaly and a large area of adhesion. Adjacent to the adhesion, a focal disruption of splenic parenchyma was found.

**Discussion:**

ASR has been reported in the literature due to infection, inflammation, mechanical and use of certain drugs. Few cases of ASR have been described due to cocaine use. Splenomegaly is one of the risk factors of ASR. The most probable mechanism of ASR in cocaine use is sudden vasoconstriction, ischemia and infarction of the parenchyma of the spleen, which can result in vascular rupture. Our case of ASR echoes the above process.

**Conclusion:**

Total splenectomy resulted in a good outcome. The patient was discharged home without any services.

## Highlights

• Spontaneous splenic rupture is a rare occurrence.
• Following cocaine used patient presented with abdominal pain and tenderness.
• Imaging study showed suspicious of splenic rupture and hemoperitoneum.
• Laparotomy showed bleeding from spleen.

## 1 Introduction

Atraumatic splenic rupture (ASR) is a rare pathological condition [1]. Cocaine induced ASR can be overlooked due to its rarity and distracting symptoms, most commonly cardio-vascular presentations, such as chest pain secondary to acute myocardial ischemia or infarction, hypertension, and arrythmias [2]. A delay in diagnosis and treatment may increase the risk of fatal outcomes. This is the case of a 23-year old male presenting with cocaine induced ASR. Our report endeavors to increase awareness of possible splenic rupture in cases of thoracoabdominal pain without a history of trauma, however, with history of cocaine use. This case report is presented in alignment with the 2018 Surgical CAse REport (SCARE) guidelines [3].

## 2 Case report

**Fig. 1:**
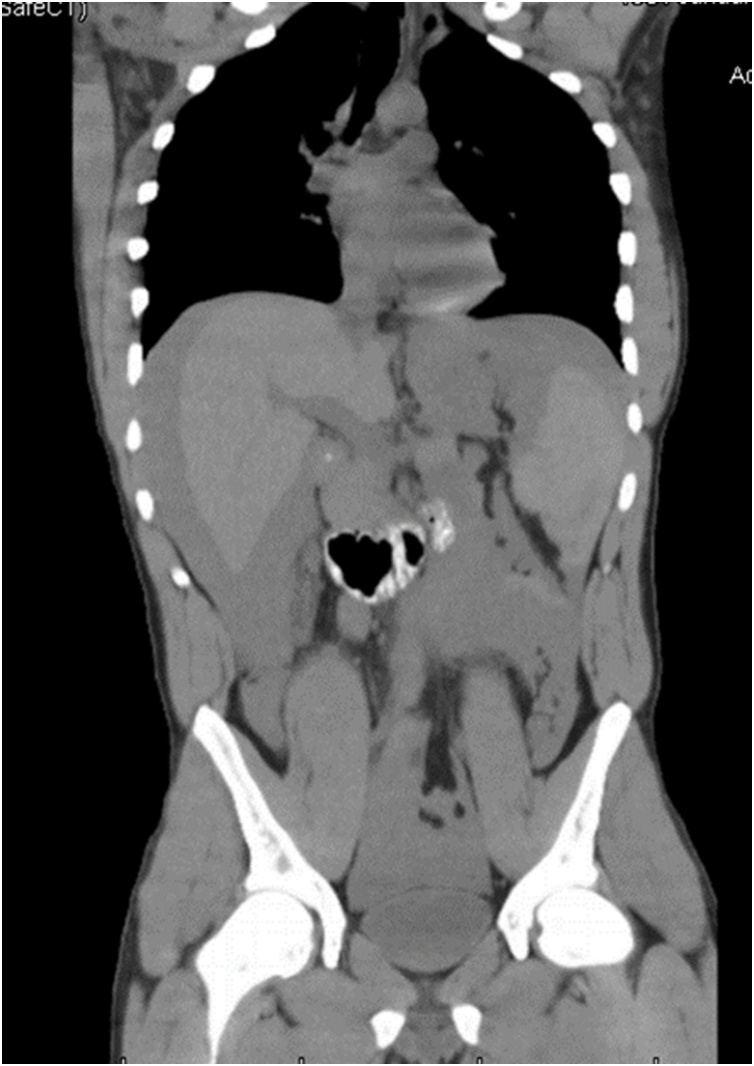
Coronal view of CT scan of chest, abdomen & pelvis.

**Fig. 2:**
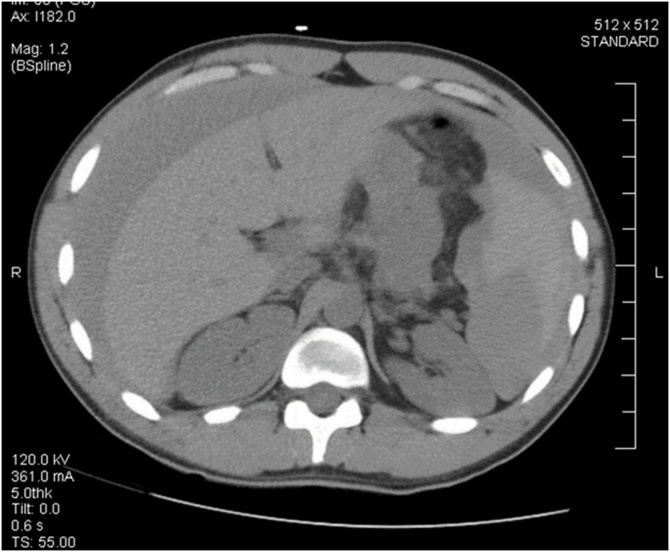
Axial view of CT scan of abdomen.

A 23-year-old male with a history of alcohol abuse and splenomegaly of unknown etiology presented with the chief complaint of abdominal pain. The patient provided information of intranasal cocaine use on the day prior to his presentation to the hospital. His pain was associated with multiple episodes of vomiting. At the initial evaluation, the patient was awake and alert with a Glasgow Coma Scale (GCS) of 15, a heart rate of 120 bpm, and a systolic blood pressure of 130 mmHg. On physical examination, the patient was found to have signs of peritonitis. Computed tomography (CT) scan imaging revealed hemoperitoneum and a possible splenic laceration (Fig. 1, Fig. 2). Hemoglobin was low at 11 mg/dL. A urine test was positive for cocaine use. The patient denied any recent history of trauma.

The patient presented with signs of peritonitis and free fluid in the abdominal cavity with a suspicion of splenic injury, and was taken to the operating room for exploratory laparotomy. Upon entering the abdomen through a midline incision, a large amount of blood was identified in the abdominal cavity. The blood was evacuated, and all four quadrants were packed with laparotomy pads. The spleen was palpated, and blood was found oozing from the hilum. Inspection from the diaphragm to pelvis revealed no other pathology. We proceeded with a splenectomy beginning with dissection of the lateral and posterior attachments to the spleen. Once the spleen was mobilized and brought to the abdominal incision, the short gastric arteries were ligated. The splenic artery and vein were then tied off with silk sutures. Smaller vessels entering the hilum were occluded with surgical clips and ligated. After all vessels were successfully divided, the spleen was removed.

The splenic bed was inspected, and hemostasis was secured. The liver was re-inspected, and the bowel ran from the ligament of Treitz to the ileocecal valve. The colon was inspected as well. All looked normal. A JP drain was left in the abdominal cavity, and the incision was closed. The patient was taken to the surgical intensive care unit (SICU) for further management.

Approximately 1.5 liters of blood were lost during surgery prompting the administration of 2 units of Packed Red Blood Cells (PRBCs) and 3.5 liters of crystalloids. An additional unit of PRBCs was transfused on post-operative day (POD) 3 to correct a low hemoglobin of 6.9 g/dl. A nasogastric (NG) tube was placed for management of ileus. On POD4, the patient was downgraded from the SICU to a medical-surgical unit. The NG tube was removed on POD 6, and the patient was encouraged to ambulate and use his incentive spirometer. On POD8, the JP drain was removed, and the patient was discharged home following post-splenectomy vaccines. During his hospital stay, differential diagnosis of infectious mononucleosis was excluded based on negative Monospot test. However, a review of the patient’s history showed that years ago the patient was positive for immunoglobulin G (IGG) titers for Epstein bar virus (EBV), which may explain the patient's past history of splenomegaly. Upon pathology review of the spleen, the spleen was 210-gram, 12.0 cm × 7.0 cm × 4.5 cm and intact. The capsule was intact and remarkable for a 4.3 cm × 1.3 cm area of adhesions on the central posterior surface adjacent to the hilum. Hilar structures were clamped. The parenchyma beneath the capsule adhesions were focally disrupted with mild accumulated blood clots. Areas of intraparenchymal hemorrhage, infarcts or mass formation were not identified. Upon microscopic evaluation, sections of the spleen showed capsular disruption with hemorrhage and adhesions.

## 3 Discussion

Splenic rupture most commonly results from thoracoabdominal trauma and leads to intraabdominal hemorrhage. The use of focused assessment with sonography in trauma (FAST) examination can detect the intraabdominal hemorrhage. While most splenic ruptures result from a traumatic mechanism, rare cases such as this exist without any attributable trauma. In such instances, the lack of a traumatic mechanism may delay the diagnosis and treatment of splenic rupture if the physician is not examining for a hemorrhage, resulting in continuous blood loss and deterioration of overall condition.

Many causes have been associated with ASR. Renzulli et al., conducted a systemic review of nearly three decades of literature comprised of 632 publications and covering nearly 850 cases of ASR. They concluded that ASR is a rare complication whose cause could be divided amongst six major groups; 1) neoplastic (acute myeloid leukemia, chronic lymphocytic leukemia, lung cancer, etc.), 2) infectious (cytomegalovirus, human immunodeficiency virus, endocarditis, etc.), 3) inflammatory (pancreatitis, rheumatoid arthritis, systemic lupus erythematosus, etc.), 4) drug related (granulocyte-colony stimulating factor, anticoagulation drugs, thrombolytic drugs, etc.), 5) mechanical (pregnancy related, congestive splenomegaly), and 6) idiopathic. The drug related group is the best etiology that fits our patient, and forms nearly 9 % of ASR. Their review continued by listing splenomegaly as one of the prognostic factors along with advanced age and neoplastic disorders, which increased the rate of ASR related mortality [1].

Cocaine’s effect on the spleen can be explained by its impact on the vasculature. Cocaine inhibits norepinephrine reuptake at nerve terminal, which results in a powerful adrenergic response as evidenced by increased heart rates and vasoconstriction [4]. The vasoconstricting properties of cocaine may cause end organ ischemia and damage [5]. Cocaine induced damage commonly manifests as angina, but the heart is not the only organ affected. Kaufman et al., showed that cocaine can reduce splenic volume by 20 % within 10 min of the drug entering the body [6]. In addition to size alteration, cocaine causes direct damage to the spleen through tissue infarction [7]. The mechanism of splenic bleeding can be due to cocaine induced vasospasm, which leads to an infarction, a sudden increase in blood pressure and vascular rupture [8,9].

Several cases have shown cocaine to be involved in ASR. A 40-year-old healthy male using cocaine intranasally developed a splenic hematoma. The patient had no history of bleeding disorders or recent infection. The proposed mechanism for the hematoma was cocaine induced vasospasm leading to infarction and hemorrhage [10]. Another healthy, 42-year-old male developed splenic rupture and hemoperitoneum following intranasal cocaine use. The mechanism is believed to be another case of cocaine induced vasospasm with subsequent splenic infarction and hemorrhage [11]. Again, a 39-year-male, with a history of alcohol abuse and deep vein thrombosis (DVT), developed a splenic rupture and nearly 1.5 liters of hemoperitoneum after intravenous cocaine use. Carlin et al. reasoned that the abrupt sympathetic activation from IV cocaine lead to vascular rupture, which affected the spleen due to large blood flow and inadequate connective tissue support [12]. Our case is unique in that our patient was younger at the time of his ASR. According to Renzulli et al, our patient met one of the three criteria for a poor outcome, splenomegaly. Though he was young and free of cancer, he did have a history of splenomegaly and this history, may have contributed to his ASR even at such a young age.

## 4 Conclusion

Traumatic mechanism is the most common cause of splenic rupture. ASR is a rare occurrence, which can result from cocaine use. Point of care physicians should be aware of ASR conditions, since prompt diagnosis and management can result in a good outcome.

## Funding

Nothing to disclose.

## Ethical approval

Since it was a single case, As per Meridian health IRB, the case report is exempted from the IRB review.

## Consent

Consent has been obtained from the patient to publish his information.

## Author contribution

Authors

1) Joshua Ramos.
2) Michael Farr.
3) SeungHoon Shin.
4) Nasim Ahmed.

## Registration of research studies

None.

## Guarantor

Nasim Ahmed.

## Provenance and peer review

Not commissioned, externally peer-reviewed.

## Declaration of Competing Interest

Nothing to disclose.
